# Comparison between ultrasound and chest X-ray to confirm central venous catheter tip position

**DOI:** 10.4102/sajr.v27i1.2587

**Published:** 2023-06-29

**Authors:** Leoni de Man, Mari Wentzel, Cornel van Rooyen, Edwin Turton

**Affiliations:** 1 Department of Anaesthesiology, Faculty of Health Sciences, University of the Free State, Bloemfontein, South Africa; 2 Department of Clinical Imaging Sciences, Faculty of Health Sciences, University of the Free State, Bloemfontein, South Africa; 3 Department of Biostatistics, Faculty of Health Sciences, University of the Free State, Bloemfontein, South Africa

**Keywords:** central venous catheter, position, chest X-ray, ultrasound, peri-operative, CVC, CXR

## Abstract

**Background:**

Mechanical central venous catheter (CVC) placement complications are mostly malposition or iatrogenic pneumothorax. Verification of catheter position by chest X-ray (CXR) is usually performed postoperatively.

**Objectives:**

This prospective observational study assessed the diagnostic accuracy of peri-operative ultrasound and a ‘bubble test’ to detect malposition and pneumothorax.

**Method:**

Sixty-one patients undergoing peri-operative CVC placement were included. An ultrasound protocol was used to directly visualise the CVC, perform the ‘bubble test’ and assess for the presence of pneumothorax. The time from agitated saline injection to visualisation of microbubbles in the right atrium was evaluated to determine the correct position of the CVC. The time required to perform the ultrasound assessment was compared to that of conducting the CXR.

**Results:**

Chest X-ray identified 12 (19.7%) malpositions while ultrasound identified 8 (13.1%). Ultrasound showed a sensitivity of 0.85 (95% confidence interval [CI]: 0.72 to 0.93) and a specificity of 0.5 (95% CI: 0.16 to 0.84). The positive and negative predictive values were 0.92 (95% CI: 0.80 to 0.98) and 0.33 (95% CI: 0.10 to 0.65), respectively. No pneumothorax was identified on ultrasound and CXR. The median time for ultrasound assessment was significantly shorter at 4 min (interquartile range [IQR]: 3–6 min), compared to performing a CXR that required a median time of 29 min (IQR: 18–56 min) (*p* < 0.0001).

**Conclusion:**

This study showed that ultrasound produced a high sensitivity and moderate specificity in detecting CVC malposition.

**Contribution:**

Ultrasound can improve efficiency when used as a rapid bedside screening test to detect CVC malposition.

## Introduction

Central venous catheterisation, first performed in 1929, became a mainstay of modern clinical practice. The central venous catheter (CVC) is an integral part of patient care in elective and emergency situations. Subsequently, CVC insertion is a routine procedure performed by anaesthetists in the peri-operative setting, and by emergency physicians and intensivists for the management of critically ill patients.

This procedure is not without risk and complications can occur during the placement of the CVC or related to the indwelling catheter itself. The main complications include incorrect position of the catheter tip, pneumothorax and arterial puncture with haematoma formation.^1^ Incorrect position of the CVC tip can lead to catheter malfunction, vessel perforation, cardiac perforation with tamponade and systemic embolism with arterial placement.^2^

Traditionally, before the ultrasound-guided technique became routine, CVC placement was performed using anatomic landmark techniques. This approach relies on two assumptions: firstly, the predicted vascular anatomy, and secondly, the patency of the vein. Unfortunately, the anatomical landmark technique cannot account for anatomic variations at the CVC insertion site.^3,4^

Research has shown a clear advantage of using the ultrasound-guided technique compared to anatomical landmarks for CVC insertion. Brass et al.^5^ reported that the use of the ultrasound-guided technique has reduced the number of complications and failed attempts, compared to the anatomical landmark technique for CVC placement in the internal jugular vein (IJV), subclavian vein (SCV) and femoral vein.^5^

**FIGURE 1:**
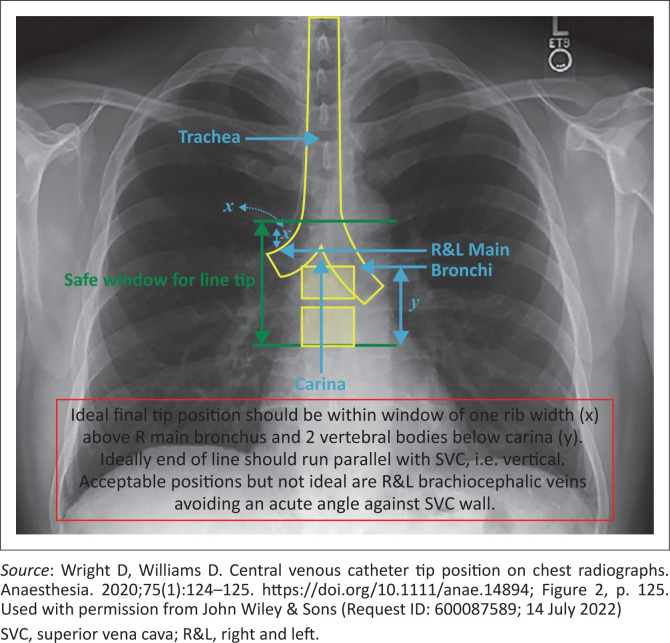
Final tip position window observed on chest X-ray (CXR).

Guidelines by the Association of Anaesthetists defined the ‘optimal’ CVC tip position ‘in the lower superior vena cava or the upper right atrium (RA)’, with the cavo-atrial junction as the lower-most acceptable point.^6^ According to Wright and Williams, the CVC tip should lie in the ‘final tip position window’ on the chest X-ray (CXR), as displayed in Figure 1.^7^

Performance of the CVC is directly related to the position of the catheter tip, as suboptimal tip position increases the risk for catheter-related complications, including dysfunction of the CVC, arrhythmia, venous thrombosis and venous erosion.^8^ These complications emphasise the importance of confirming the tip position before using the CVC. It is best practice to confirm the correct placement of the CVC before using the catheter. The standard CXR is still considered the gold standard to locate the CVC tip to exclude malposition and to rule out immediate complications such as pneumothorax.^9^

Although widely used to confirm CVC positioning, the reliability of CXRs is not absolute. The accuracy of the CXR in locating the catheter tip is overestimated as the interpretation of a CXR is subjective, and interpretation errors may occur according to the training level and experience of clinicians, subjecting it to interobserver variability.^10,11^ The entire procedure of performing a CXR, from initial request to final report, is time consuming. If malposition of the CVC is detected on the CXR, it needs to be repositioned or possibly re-inserted, causing a potentially detrimental delay in the care of a critically ill patient. Furthermore, repeating the CXR to confirm correct positioning increases both the dose of radiation exposure and the cost involved.^9,12,13^ It is also not a feasible option in the theatre setting during the peri-operative period.

The use of bedside ultrasonography to confirm the position of the CVC and rule out pneumothorax may be an acceptable alternative in theatre and in the intensive care unit (ICU). Using ultrasound combined with a saline flush or ‘bubble test’ to determine the CVC tip position was described by Vezzani et al.^14^ A rapid injection of 5 mL of agitated saline, comprising 90% saline and 10% air, as a bolus through the catheter, results in a stream of microbubbles seen in the RA through the tip, confirming the tip position.^14^ The ‘push-to-bubbles’ time was measured to classify the position of the CVC tip. Vezzani et al. classified and interpreted the test as represented in Table 1. They showed a 96% sensitivity and 93% specificity in detecting catheter malposition using the agitated saline flush test. They found that the mean time required to perform ultrasonography plus contrast-enhanced ultrasonography was 10 ± 5 min opposed to 83 ± 79 min for a chest radiograph (*p* < 0.05).^14^

**TABLE 1:** Interpretation of the ‘bubble test’.

Observation	Interpretation
No bubbles	Negative test: an aberrant or too distal tip
Few bubbles or appearance in > 2 s	Negative test: possible misplacement (probably in SCV or IJV)
Numerous bubbles with turbulent flow from RA within 2 s	Negative test: intra-atrial position
Numerous bubbles with linear flow from SVC within 2 s	Positive test: correct position

*Source*: Vezzani A, Brusasco C, Palermo S, Launo C, Mergoni M, Corradi F. Ultrasound localization of central vein catheter and detection of postprocedural pneumothorax: An alternative to chest radiography. Crit Care Med. 2010;38(2):533–538. https://doi.org/10.1097/CCM.0b013e3181c0328f

IJV, internal jugular vein; SCV, subclavian vein; RA, right atrium.

The ‘bubble test’ is used in the field of cardiology to evaluate for the presence of right to left intracardiac shunts. Case reports suggest that the ‘bubble test’ rarely causes ischaemic complications such as stroke or transient ischaemic attack. Romero et al. reported five cases of cerebral ischaemic events occurring immediately or within 5 min of the ‘bubble test’ in 3314 patients.^15^

When using the anatomical landmark-guided technique for CVC insertion, the incidence of pneumothorax after placement in the IJV is 0.3% – 1.0% and 1.6% – 2.3% when placed in the SCV. With the ultrasound-guided technique, the incidence of pneumothorax was reduced, and lung ultrasound showed a higher sensitivity than CXR in detecting a pneumothorax.^16,17^

The advantages of bedside ultrasonography over CXR after CVC insertion include decreased use of hospital resources, reduced radiation exposure and a decrease in the time delay from insertion to full functioning of the CVC.^18^ Globally, several institutions have studied the ultrasound technique to confirm the results and assess whether it would be a viable option to determine CVC tip position. Several systematic reviews and meta-analyses reported that ultrasound can be used as an accurate diagnostic modality to detect CVC tip malposition and pneumothorax.^18,19,20^

In this study, the authors compared the accuracy of bedside ultrasound with a CXR to confirm peri-operatively the position of the CVC tip in surgical patients at Universitas Academic Hospital (UAH) in Bloemfontein, South Africa. Currently, no literature is available on this topic in South Africa, and by implementing the study, it might show that ultrasound could be used as a quick, easy, and feasible measure in the hospital setting to confirm CVC tip placement and detect pneumothorax resulting from the procedure.

The primary aim of the study was to prove that bedside ultrasonography would accurately determine the position of the CVC tip and that performing a bedside ultrasound would be more time efficient than a CXR. The secondary objectives included the detection of pneumothorax with bedside ultrasonography and compiling a demographic profile of patients receiving a CVC.

## Methods

### Study design and sampling

A prospective, observational, cross-sectional, analytical study was performed. A CVC was inserted under ultrasound guidance in the peri-operative period. The position of the catheter tip was determined by using ultrasound with the ‘bubble test’ and was compared to a postoperative CXR.

The study population included all patients ≥ 18 years of age booked for elective surgery at UAH between 01 March 2020 and 31 December 2020, in which the placement of a CVC was clinically indicated as part of the anaesthetic management. The study sample was selected according to specific inclusion and exclusion criteria, although it was subjected to convenience sampling.

### Measurement protocols

#### Central venous catheter placement

The CVC was placed in either the right or left IJV or the right or left SCV. The position and depth of the CVC were dictated by patient and surgical factors. The CVC was inserted under sterile conditions with ultrasound guidance using the Seldinger technique.^21^ The CVCs that were used were the Arrow 7 French Two-Lumen CVC Set with Blue FlexTip^®^ (SKU/Article # CA27702; Teleflex Incorporated; Morrisville, North Carolina (NC), United States [US]) of varying length between 16 cm (product code CV-12702) and 20 cm (product code CV-16702-SA2).

#### Ultrasound and ‘bubble test’

A vascular examination was done where bilateral IJVs and SCVs were scanned with the high-frequency linear probe to directly visualise the CVC passing either proximally or distally to the site of puncture, or in any vessel not used for the CVC insertion. When the CVC was visualised proximal to the puncture site or in another vessel not used for the insertion, it was classified as a malposition.

The ipsilateral lung field of the site of the CVC insertion was then scanned with the high-frequency linear probe to diagnose or exclude an iatrogenic pneumothorax. The presence of lung sliding rules out a pneumothorax. A pneumothorax is highly suspected if lung sliding is absent and identification of the lung point using M-mode is 100% specific for diagnosing a pneumothorax.^22^

A cardiac examination was performed with the RA visualised from the apical four-chamber or subcostal views using the cardiac probe. The presence of the tip of the CVC in the RA was classified as a malposition as it was intra-atrial and thus too deep.

**FIGURE 2:**
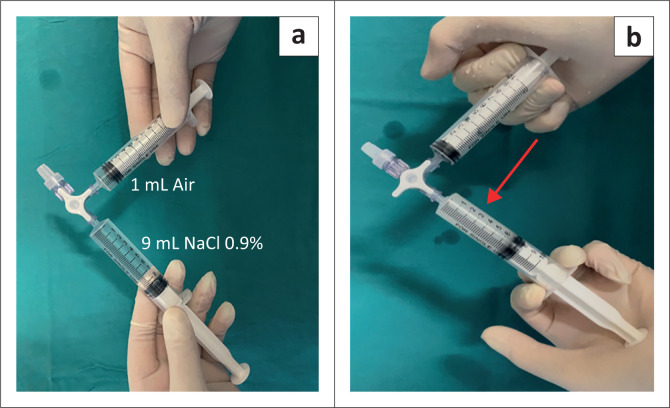
(a) Two syringes connected with a three-way stopcock. One syringe filled with 90% normal saline and the other syringe filled with 10% air. (b) The saline and air are rapidly exchanged between the two syringes by way of the three-way stopcock to produce microbubbles as indicated by the arrow.

**FIGURE 3:**
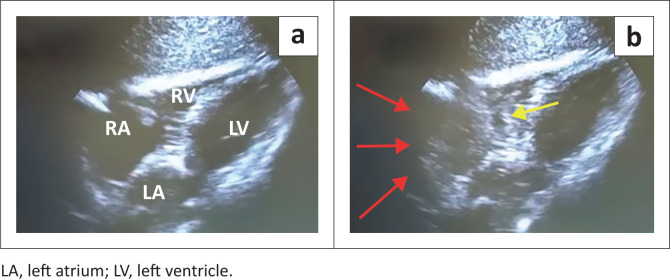
(a) Subcostal ultrasound view of the heart. The empty right atrium (RA) and right ventricle (RV) at the start of the ‘bubble test’. (b) Microbubbles visible within the RA (red arrows) and leading into the RV (yellow arrow) during the ‘bubble test’ at the 1-s mark, indicating correct CVC tip position.

The ‘bubble test’ involved an injection of agitated saline into the distal lumen of the CVC. The agitated saline was mixed by an exchange of 90% saline and 10% air between two syringes by way of a three-way stopcock, as illustrated in Figure 2. A rapid bolus of 5 mL of the agitated saline mixture was administered over 2 s and repeated if necessary. A video clip of the RA was recorded digitally during the ‘bubble test’. This video clip was later reviewed to determine the time interval from injection to visualisation of the microbubbles in the RA. An example of the ultrasound images is displayed in Figure 3. The ‘bubble test’ was interpreted according to the criteria for classifying the position of the CVC tip (Table 1).^14^ The time required to complete the ultrasound and ‘bubble test’ was recorded on the data sheet.

#### Chest X-ray

A mobile CXR was performed postoperatively. The images were analysed by a registrar in her second year of postgraduate training in the Department of Clinical Imaging Sciences at UAH. The position of the CVC tip was then classified as either correctly positioned or malpositioned, as defined by Wright and Williams^7^ (Figure 1). The presence or absence of a pneumothorax was reported. The time lapse from CXR requisition to the availability of the images on the imaging system was also recorded.

### Data collection and analysis

A pilot study was conducted on five patients to test the measurement protocol. No changes to the data sheet were required, and the pilot study results were included in the main analysis. All data and demographic information were recorded on an individual data sheet for each patient. The data were then combined into one password-protected Microsoft Excel spreadsheet to facilitate the analysis, which was conducted by the Department of Biostatistics, University of the Free State.

From the data obtained, the following analyses were performed: (1) the accuracy of bedside ultrasonography with the ‘bubble test’ compared to CXR to confirm CVC tip placement; (2) the accuracy of bedside ultrasonography compared to CXR to detect a pneumothorax and (3) comparison of the average amount of time required to complete the ultrasound protocol versus obtaining a CXR.

Numerical variables were summarised by medians, minimum, maximum and interquartile range (IQR). Categorical variables were summarised by frequencies and percentages. Within-group changes were evaluated using the signed-rank test for numeric paired data (non-parametric data). The Shapiro-Wilk statistic was used for assessing the normality of the data. The 95% CI for the median difference between ultrasound and CXR was also calculated. The analysis was done by the Department of Biostatistics, using SAS Version 9.4 (SAS Institute, Inc.; Cary, North Carolina, US).

### Ethical considerations

Approval for the study was obtained from the Health Sciences Research Ethics Committee (HSREC) of the University of the Free State (reference number UFS-HSD2019/1531/2403) and the Free State province Department of Health (reference number FS_201911_001). Each participant received an information document and informed consent was obtained before inclusion in the study. The patients’ information was kept confidential.

## Results

**FIGURE 4:**
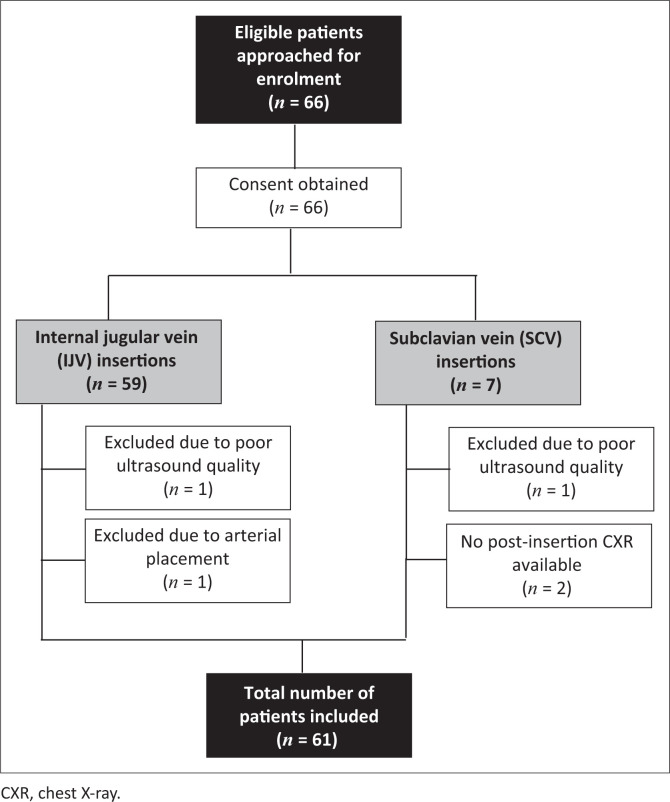
Flow diagram illustrating the enrolment process.

In total, 66 patients were approached for enrolment in the study from 01 March 2020 to 31 December 2020 at UAH. The enrolment process is illustrated in Figure 4. Five patients were excluded from the final data analyses. Bilateral vascular examinations of the IJVs and SCVs with ultrasound were feasible in 100% of cases. The feasibility rate to obtain adequate imaging of the RA was 96.7%. However, 30 (49.2%) of the 61 patients were either overweight or obese. In one patient, no microbubbles were visualised in the RA. The CVC was most likely placed inadvertently in the artery, which was confirmed by comparison of blood gasses taken from the CVC and the arterial line. The CVC was removed and replaced with a femoral vein CVC and the patient was excluded from the study.

Table 2 shows the demographic characteristics of the patients. The median age of the 61 participants was 50 years, with an IQR of 42–61 years. The median body mass index (BMI) was 24.8 kg/m^2^ (IQR: 21.3 kg/m^2^ – 32.5 kg/m^2^).

**TABLE 2:** Demographic characteristics of the patients and central venous catheter placement (*n* = 61).

Variable	*n*	%
**Gender**		
Male	41	67.2
Female	20	32.8
**Body mass index (BMI) category**		
Underweight (≤ 18.49 kg/m^2^)	6	9.8
Normal (18.5 kg/m^2^ – 24.99 kg/m^2^)	25	41.0
Overweight (25.0 kg/m^2^ – 29.99 kg/m^2^)	12	19.7
Obese (≥ 30 kg/m^2^)	18	29.5
**Site of CVC insertion**		
Internal jugular vein (IJV)	57	93.4
Subclavian vein (SCV)	4	6.6
**Side of CVC insertion**		
Right	59	96.7
Left	2	3.3

CVC, central venous catheter.

During the analysis of the results, the authors used CXR as the gold standard to which the authors compared the ultrasound technique. The authors defined a ‘true-positive’ result as an ultrasound ‘bubble test’ showing the correct CVC tip position confirmed as correct by the CXR and a ‘true-negative’ result as a malpositioned catheter tip on both CXR and ultrasound. A two-by-two table showing the true- and false-positive and true- and false-negative results is included (Table 3).

**TABLE 3:** Two-by-two table showing true-positive and false-positive and negative results of the comparison between ultrasound with ‘bubble test’ and chest X-ray.

Ultrasound with ‘bubble test’	CXR	
	Correct position (positive)	Incorrect position (negative)
**Correct position (positive)**	True-positive	False-positive
*n*	45	8
%	73.8	13.1
**Incorrect position (negative)**	False-negative	True-negative
*n*	4	4
%	6.6	6.6

CXR, chest X-ray.

Based on these findings, ultrasonography showed a sensitivity of 0.85 (95% CI: 0.72 to 0.93) and a specificity of 0.5 (95% CI: 0.16 to 0.84). The positive and negative predictive values were 0.92 (95% CI: 0.80 to 0.98) and 0.33 (95% CI: 0.10 to 0.65), respectively. Cohen’s kappa was 0.29 (95% CI: –0.01 to 0.59), which indicated fair agreement.

Lung ultrasound was performed on all the patients included in the study. No pneumothoraces were identified on ultrasound or CXR. The entire ultrasound protocol took a median of 4 min to complete (IQR of 3–6 min), with a maximum completion time of 18 min. In comparison, the median time required for the CXR was 29 min (IQR: 18–56 min), with a maximum completion time of 204 min. The 95% CI for the median difference in completion time between the ultrasound and the CXR was –36 to –17, which was statistically significant (*p* < 0.0001).

## Discussion

The results show that ultrasound is an excellent test to confirm the correct placement of a CVC, but it is less than perfect to rule out malposition. A high false-positive rate was observed because of the low specificity. In eight false-positive cases, ultrasound did not reveal any abnormalities during the vascular examination, although CVC placement was classified as malpositioned on CXR. However, ultrasound was excellent at detecting a correct catheter tip position, as reflected by the high sensitivity rate.

This study produced an excellent positive predictive value to detect the correct catheter tip position, keeping in mind the low incidence of catheter tip malpositions and moderate specificity. However, the results showed a poor negative predictive value, which could have been related to the low incidence of catheter tip malpositions or true-negative cases.

Interobserver variability in detecting tip malposition on CXR and poor quality CXR images may result in an incorrect high false-positive rate. This could account for the difference in specificity when comparing this study’s results to those of Vezzani et al.^14^

In a recent prospective multicentre study,^23^ one of the primary outcomes was the diagnostic accuracy of ultrasound to detect CVC malposition. Among 758 CVC placements evaluated, malposition occurred in 3.3% (*n* = 25) of the cases, showing that ultrasound had a sensitivity of 0.70 (95% CI: 0.49 to 0.86) and specificity of 0.99 (95% CI: 0.98 to 1.00). However, they defined a ‘true-positive’ result as a CVC malposition identified on ultrasound and then confirmed as a malposition by CXR and a ‘true-negative’ result as a correctly positioned CVC tip identified on both CXR and ultrasound. Only seven false-negative and five false-positive outcomes were identified among the 758 CVC placements evaluated in this study.^23^ If the authors defined a ‘true-positive’ and a ‘true-negative’ result as in the study by Smit et al.^23^ and re-analysed the data, the sensitivity and specificity would be 0.33 and 0.92, respectively. Table 4 shows how the sensitivity and the specificity of three different studies^24,25,26^ compared to the findings in this study. These studies defined a ‘true-positive’ and ‘true-negative’ result similar to the definition used in this study.

**TABLE 4:** Comparison of previously published findings on the ‘bubble test’ (ultrasonography) to confirm central venous catheter tip position.

Study	Year	Patients (*n*)	Sensitivity	Specificity
Weekes et al.^25^	2016	151	1.00	0.75
Kamalipour et al.^26^	2016	116	0.98	0.69
Blans et al.^27^	2016	53	0.98	-
Current study	2020	61	0.85	0.50

*Source:* Please see the full reference list of the article for more information

Smit et al. demonstrated a clinically relevant incidence of CVC malposition in 5 (0.7%) of the 758 patients, with a clinically relevant catheter malposition being regarded as when the CVC needed to be re-inserted or caused a complication.^23^ This specific analysis was not done in this study, but it might provide valuable clinical information if included in future research.

The limit of 2 seconds to visualise the microbubbles in the RA might be a rather arbitrary value. According to the Hagen-Poiseuille equation,^27^ the flow of the agitated saline mixture through the CVC depends on the pressure difference, fluid viscosity, catheter length and diameter. Consequently, shorter central lines with larger diameters can have significantly increased flow and the agitated saline mixture will reach the RA faster. A higher cardiac output can also result in the agitated saline mixture reaching the RA more rapidly and the 2-s cut-off value could produce a false-negative result. Therefore, a higher sensitivity could be produced if a duration of less than 2 s were accepted. However, visualisation of microbubbles in the RA within 2 s confirms intravenous CVC position, implying an unimpaired fast delivery of medication into the RA.^23^ Future studies can assess the clinical significance of these varying lengths of the CVC on the occurrence of flow and time from injection to visualisation of microbubbles in the RA.

One patient had an inadvertent arterial placement of the CVC that was detected by the ‘bubble test’. The CVC would have been used during the surgery if no other method to confirm correct CVC tip positioning was used intra-operatively. These observations emphasise the importance of the ‘bubble test’ to detect CVC tip malposition.

There were no complications related to the ‘bubble test’ in this study. The available data were not sufficient to estimate the incidence of such events, although such complications appear to be rare, as reported by Romero et al.,^15^ confirming the excellent safety profile of the ‘bubble test’.

Ultrasound and CXR showed 100% agreement in detecting pneumothorax ipsilateral to the site of CVC insertion, but this study was limited by a small sample size. It is known that pneumothorax is an iatrogenic complication of CVC insertion. The incidence of pneumothorax varies. Smit et al. reported their incidence of pneumothorax according to CXR and ultrasound as 0.7% (*n* = 5) and 1.5% (*n* = 11), respectively, among a total of 758 patients.^23^ In a recent prospective multicentre cohort study, Adrian et al. reported pneumothoraces in 17 (0.1%) of 12 066 patients that had a CVC inserted above the diaphragm.^28^ However, there is no indication of exactly when a pneumothorax will develop after a pleural puncture, especially in patients receiving mechanical positive pressure ventilation. Cases have been reported of patients developing a tension pneumothorax immediately after attempting a CVC, but this may also be delayed and does not present immediately.^29,30,31^

Rowan et al.^32^ compared the accuracy of ultrasonography with that of a mobile supine CXR to detect traumatic pneumothoraces in 27 patients who sustained blunt chest trauma and then used computed tomography (CT) as the gold standard for confirmation. Supine CXRs had a sensitivity of 36% in detecting pneumothorax and were regarded as unreliable in making the diagnosis. Ultrasonography was found to be more sensitive than the mobile CXR and showed a sensitivity similar to CT in the detection of pneumothoraces.^32^ During the development of a pneumothorax in a supine patient, the air initially disperses within the nondependent and medial parts of the chest and can therefore be invisible on a mobile supine CXR. A pneumothorax may only be detected on CXR once the air volume extends to the apical and lateral parts of the chest.^33^ Based on current best practice, a CXR should be performed immediately after a CVC insertion, although it is possible that there might not be adequate time for a sufficiently large pneumothorax to develop and be detected on a supine CXR. This scenario further highlights the importance of point-of-care (POC) ultrasound to detect a pneumothorax, as ultrasonography can be employed immediately when a pneumothorax is suspected. Based on the results presented, an ultrasound is quicker to perform than a mobile CXR, and therefore, no time delay will occur from detection until treatment of the pneumothorax.

Two limitations of this study coincidentally were the two modalities investigated, namely CXR and ultrasonography. As already noted, the interpretation of a CXR is subjective, and consequently, errors may occur. Substantial interobserver variability may occur because of the training level and experience of clinicians,^10,11^ which can also be influenced by the image quality of the CXR. The postoperative CXRs were mobile radiographs in the supine or semi-recumbent position, and it is known that mobile CXRs are of an inferior quality. In a case report by Sharma et al., they described how the CVC tip changed position from the level of the 5th rib in the semi-sitting position to the level between the 6th and 7th ribs in the supine position.^34^ All these factors will influence the interpretation of the tip position. This might account for the eight (13.1%) false-positive cases identified in this study, in which CVC malpositions were seen on the CXR, but ultrasound did not reveal any abnormalities during the vascular examination. However, these adverse events might call for a change from CXR being the gold standard. In this study, the evaluation and interpretation of the CXR were performed by a single operator, which eliminated bias and improved the strength of the study.

Ultrasound has similar shortcomings. Again, image quality of the RA on ultrasound will affect visualisation of the microbubbles and interpretation of the ‘bubble test’. Logically, when the RA cannot be adequately observed on ultrasound, the ‘bubble test’ will not be a feasible method to determine correct CVC tip positioning.

In our setting, the ultrasound with the ‘bubble test’ method is not routinely used to determine CVC tip positioning. Consequently, the technique must be taught to inexperienced colleagues. Tran et al.^35^ performed a survey among members of the College of Emergency Physicians and the Society of Critical Care Medicine to determine their perception of ultrasound for the confirmation of above-diaphragm CVC placement. Of the 136 participants, 31% would use ultrasound only for CVC confirmation, while 42% were confident with performing ultrasound for this purpose.^30^

Consequently, interobserver variability may also occur when initiating this practice because of the lack of training and inexperienced clinicians. This interobserver variability may improve when the technique is performed more regularly. In this study, a single operator performed and interpreted the ‘bubble test’, which contributed to the strength of the study as interobserver variability was eliminated. In 2018, Korsten et al.^36^ investigated the performance of medical residents after limited training on the ‘bubble test’ to assess CVC tip position. Their study represented routine clinical situations where physicians with different levels of experience were performing the ultrasound examinations. All the participants were familiar with using vascular ultrasound during CVC placement, but all had either very little or no experience with cardiac ultrasound before being trained. Their results showed good interobserver agreement, suggesting that the ‘bubble test’ could be equally well performed after a training session and that all the participants were able to identify CVC malposition.^36^

Other limiting factors include the small study sample size, the site of CVC insertion, the use of different ultrasound machines and the image settings that were not standardised. Only 61 patients were included in the final data analyses and most of these CVCs were inserted in the right IJV based on operator preference, and patient- and surgery-related factors. A larger study sample with a more equal distribution between CVC insertion sites and standardisation of the ultrasound equipment might yield different results.

Transoesophageal echocardiography (TOE) and intracavitary electrocardiogram-guided CVC placement are other means to confirm tip position. Although TOE has been shown to be the most accurate method to verify tip location, it is costly, invasive, cannot be used routinely and cannot be used to exclude the presence of a pneumothorax.^37^ Intracavitary electrocardiogram-guided CVC placement provides a real-time, accurate tip position confirmation during the insertion procedure. This tip tracking technology uses magnetic or electromagnetic methods to track the movement of the catheter tip. Unfortunately, not all systems can be used with all the various CVC types available. Some manufacturers limit their systems to be used exclusively with their products, limiting the use of this method of confirmation in certain hospital settings as they may not have access to these specific products.^38^ This method appears to be a suitable replacement for post-procedural CXR confirmation as it has been proven to be safe and accurate. As with TOE, this technique cannot be used to exclude the presence of a pneumothorax. Consequently, ultrasound remains the most suitable option from the other options available to confirm CVC tip position and exclude pneumothorax.

Future studies in this field may focus on comparing the accuracy of ultrasound with the ‘bubble test’ for the detection of CVC tip position to direct visualisation of the CVC tip using TOE. This direct visualisation of the CVC tip with TOE can also be compared with the CVC tip interpretation on CXR, specifically when a mobile CXR is performed. This might contribute to consensus on the accuracy of ultrasound to detect CVC tip malposition, as some of these limitations may be eliminated. Comparative cost analysis of ultrasound versus CXR for CVC confirmation is another area of potential research.

## Conclusion

Ultrasonography with the ‘bubble test’ produced a high sensitivity and moderate specificity to detect CVC malposition and can be used as a rapid bedside screening test to evaluate CVC placement. Although training in this technique is necessary, the use of ultrasound to ascertain the correct CVC tip position and the absence of pneumothorax will result in improved efficiency in the hospital setting, reduced radiation exposure to the patient, quicker detection and treatment of complications and subsequently improved patient outcomes.
